# A phosphodiesterase 4-controlled switch between memory extinction and strengthening in the hippocampus

**DOI:** 10.3389/fnbeh.2014.00091

**Published:** 2014-03-17

**Authors:** Rafael Roesler, Gustavo K. Reolon, Natasha Maurmann, Gilberto Schwartsmann, Nadja Schröder, Olavo B. Amaral, Samira Valvassori, João Quevedo

**Affiliations:** ^1^Laboratory of Neuropharmacology and Neural Tumor Biology, Department of Pharmacology, Institute for Basic Health Sciences, Federal University of Rio Grande do SulPorto Alegre, Brazil; ^2^Cancer Research Laboratory, University Hospital Research Center (CPE-HCPA), Federal University of Rio Grande do SulPorto Alegre, Brazil; ^3^National Institute for Translational Medicine (INCT-TM)Porto Alegre, Brazil; ^4^Department of Internal Medicine, School of Medicine, Federal University of Rio Grande do SulPorto Alegre, Brazil; ^5^Neurobiology and Developmental Biology Laboratory, Faculty of Biosciences, Pontifical Catholic UniversityPorto Alegre, Brazil; ^6^Leopoldo de Meis Institute of Medical Biochemistry, Federal University of Rio de JaneiroRio de Janeiro, Brazil; ^7^Laboratory of Neurosciences, Graduate Program in Health Sciences, Academic Unit of Health Sciences, University of Southern Santa Catarina (UNESC)Criciúma, Brazil; ^8^Center for Experimental Models in Psychiatry, Department of Psychiatry and Behavioral Sciences, The University of Texas Medical School at HoustonHouston, TX, USA

**Keywords:** phosphodiesterase 4, rolipram, extinction, reconsolidation, hippocampus, inhibitory avoidance, fear memory

## Abstract

Established fear-related memories can undergo phenomena such as extinction or reconsolidation when recalled. Extinction probably involves the creation of a new, competing memory trace that decreases fear expression, whereas reconsolidation can mediate memory maintenance, updating, or strengthening. The factors determining whether retrieval will initiate extinction, reconsolidation, or neither of these two processes include training intensity, duration of the retrieval session, and age of the memory. However, previous studies have not shown that the same behavioral protocol can be used to induce either extinction or reconsolidation and strengthening, depending on the pharmacological intervention used. Here we show that, within an experiment that leads to extinction in control rats, memory can be strengthened if rolipram, a selective inhibitor of phosphodiesterase type 4 (PDE4), is administered into the dorsal hippocampus immediately after retrieval. The memory-enhancing effect of rolipram lasted for at least 1 week, was blocked by the protein synthesis inhibitor anisomycin, and did not occur when drug administration was not paired with retrieval. These findings indicate that the behavioral outcome of memory retrieval can be pharmacologically switched from extinction to strengthening. The cAMP/protein kinase A (PKA) signaling pathway might be a crucial mechanism determining the fate of memories after recall.

## Introduction

Newly formed memory traces become increasingly resistant to disruption or enhancement by different types of interference, through the process known as *consolidation* (McGaugh, 2000). However, the retrieval of a previously consolidated memory can lead to phenomena such as *extinction*, which is likely based on the formation of a new memory that weakens the expression of the original learning (Bouton and Bolles, 1979; Quirk and Mueller, 2008), and *reconsolidation*, a process involving labilization followed by a new phase of stabilization, that may serve to maintain, update, or strengthen the memory trace (Nader et al., 2000; Sara, 2000a; Alberini, 2011). Extinction and reconsolidation are usually viewed as two opposing and possibly competing processes triggered by retrieval, resulting in long-lasting modifications of the original memory trace, or at least of its behavioral expression.

The factors determining whether extinction, reconsolidation accompanied by strengthening, or neither of these processes will be initiated by retrieval remain poorly understood. Studies have found that manipulations of training intensity, retrieval duration, and age of the memory can be used to guide memory retrieval towards extinction or reconsolidation. For example, the use of longer retrieval sessions led to extinction, while a shorter exposure to the learning environment during retrieval induces labilization and sensitivity to drug interference (Pedreira and Maldonado, 2003; Suzuki et al., 2004; Lee et al., 2006). In addition, retrieval more likely results in reconsolidation-mediated strengthening when the original memory is younger or more robust (Eisenberg et al., 2003; Inda et al., 2011). Thus, some of the behavioral training and testing conditions that allow for the discrimination between extinction and reconsolidation have been characterized.

However, previous studies have not shown whether purely pharmacological, biochemical, or molecular factors can act as switches determining the occurrence of extinction or reconsolidation upon retrieval. Here we investigated the effect of post-retrieval phosphodiesterase type 4 (PDE4) inhibition in the dorsal hippocampus on memory retention. The original aim of this study was to examine the role of PDE4 in extinction, and our initial hypothesis was that rolipram could accelerate extinction of inhibitory avoidance (IA). We chose rolipram as a selective PDE4 inhibitor known to enhance hippocampal long-term potentiation (LTP) and memory in different models (Barad et al., 1998; Tully et al., 2003). Surprisingly, we found that, under experimental conditions in which retrieval normally leads to extinction, this outcome can be switched to memory strengthening by a single intrahippocampal infusion of rolipram. To our knowledge, this finding provides the first evidence that whether retrieval will lead to extinction or strengthening (possibly mediated by reconsolidation) can be influenced by manipulating cell signaling mechanisms in the brain.

## Methods

### Animals

Adult male Wistar rats (310–400 g of weight, around 90 days of age at time of surgery) were obtained from the institutional breeding facility (CREAL, ICBS, UFRGS, Porto Alegre, Brazil) and the State Health Science Research Foundation (FEPPS-RS, Porto Alegre, Brazil). Animals were housed five per cage in plastic cages with sawdust bedding, and maintained on a 12 h light/dark cycle at a room temperature of 22 ± 1 C. The rats were allowed *ad libitum* access to standardized pellet food and water. All experiments took place between 9 AM and 6 PM. All experimental procedures were performed in accordance with the National Institutes of Health (NIH) Guide for the Care and Use of Laboratory Animals and were approved by the institutional animal care committee (CEUA-HCPA 05-519).

### Surgery

Animals were implanted under anesthesia with ketamine (75 mg/kg) and xylazine (25 mg/kg) with bilateral 14-mm or 9.0-mm, 23-gauge guide cannulae aimed 1.0 mm above the CA1 area of the dorsal hippocampus, as described in previous studies (Roesler et al., 2006; Jobim et al., 2012). Coordinates anteroposterior, −4.3 mm from bregma; mediolateral, ±3.0 mm from bregma; ventral, −2.0 mm from skull surface) were obtained from the atlas of Paxinos and Watson (2007). Animals were allowed to recover for at least 7 days after surgery.

### Drugs and infusion procedures

The general procedures for intra-hippocampal infusions were as described in previous reports (Quevedo et al., 1999; Luft et al., 2006; Roesler et al., 2006). At the time of infusion, a 30-gauge infusion needle was fitted into the guide cannula. The tip of the infusion needle protruded 1.0 mm beyond the guide cannula and was aimed at the CA1 area of the dorsal hippocampus. The animals received, via the infusion cannula, a bilateral 0.8 μl infusion of vehicle (20% dimethylsulfoxide, DMSO, in saline), the PDE4 inhibitor rolipram (7.5 μg /side dissolved in vehicle; Sigma-Aldrich, St. Louis, USA), the protein synthesis inhibitor anisomycin (80.0 μg/side dissolved in vehicle; Sigma-Aldrich, St. Louis, USA), or rolipram combined with anisomycin at the doses described above. Drug doses were chosen on the basis of previous studies (Quevedo et al., 1999; Vianna et al., 2001; Luft et al., 2006; Werenicz et al., 2012). Drug or vehicle was infused over a 30-s period. Solutions were freshly prepared before each experiment.

In different experiments, intra-hippocampal infusions were given immediately after the first retrieval session (which also served as extinction training), 1 h after retrieval (delayed infusion controls), 24 h after training in the absence of retrieval (no retrieval controls), or immediately after training.

### Inhibitory avoidance

We used the single-trial step-down IA task as an established model of fear memory. In step-down IA training, animals learn to associate a location in the training apparatus (a grid floor) with an aversive stimulus (footshock). The general procedures for IA behavioral training and retention tests have been described in previous reports (Quevedo et al., 1999; Luft et al., 2006; Jobim et al., 2012). The IA apparatus was a 50 × 25 × 25-cm acrylic box (Albarsch, Porto Alegre, Brazil) whose floor consisted of parallel caliber stainless steel bars (1 mm diameter) spaced 1 cm apart. A 7-cm wide, 2.5-cm high platform was placed on the floor of the box against the left wall.

On training trials, rats were placed on the platform and their latency to step down on the grid with all four paws was measured with a digital chronometer connected to the box control unit. Immediately after stepping down on the grid, rats received a mild footshock (0.5-mA, 2.0-s) and were removed from the apparatus immediately afterwards. Retention test trials (retrieval sessions also serving as extinction training trials) took place at different time points after training by placing the rats on the platform and recording their latencies to step down. No footshock was presented during retention test trials. In trials in which post-retrieval drug infusions were given, rats that did not step down to the grid floor within 180 s were led by the experimenter to step down on the grid floor for 3 s. Step-down latencies on the retention test trial (maximum 180 s) were used as a measure of IA memory retention. In some of the experiments, rats showing extinction were given a 0.2-mA, 2.0-s reminder footshock at the end of the series of testing sessions (Tronel and Alberini, 2007), followed by an additional retention test 24 h later. It should be mentioned that this is a collaborative experiment in which two identical IA training apparatuses in two different laboratories were used for different experiments.

### Histology

Twenty-four to 72 h after behavioral testing, a 0.5-μl infusion of a 4% methylene blue solution was given into the dorsal hippocampus. Rats were sacrificed by decapitation 15 min later, and their brains were removed and stored in 10% formalin for at least 72 h. The brains were sectioned and examined for cannulae placement in the hippocampus. The extension of the methylene blue dye was taken as indicative of diffusion of the drugs given to each rat. Animals included in the final analysis (146 rats) had bilaterally placed cannula in the intended sites. Infusion placements into the dorsal hippocampus, as revealed by the diffusion of methylene blue, was similar to those described in previous reports (Quevedo et al., 1999; Roesler et al., 2006, 2009; Jobim et al., 2012; data not shown).

### Statistics

Data are shown as mean ± S.E.M. retention test latencies to step-down (s). Comparisons of training and retention test step-down latencies between groups were performed using Kruskal-Wallis analysis of variance followed by Mann-Whitney *U*-tests, two-tailed, when appropriate. Comparisons between behavioral sessions within the same group were made using Friedman tests. Nonparametric tests were chosen because of the ceiling cutoff imposed to retention test latencies (Quevedo et al., 1999; Vianna et al., 2001; Luft et al., 2006; Roesler et al., 2006; Jobim et al., 2012). In all comparisons, *P* < 0.05 was considered to indicate statistical significance.

## Results

### Administration of rolipram into the dorsal hippocampus after retrieval switches memory from extinction to strengthening

**Figure 1:**
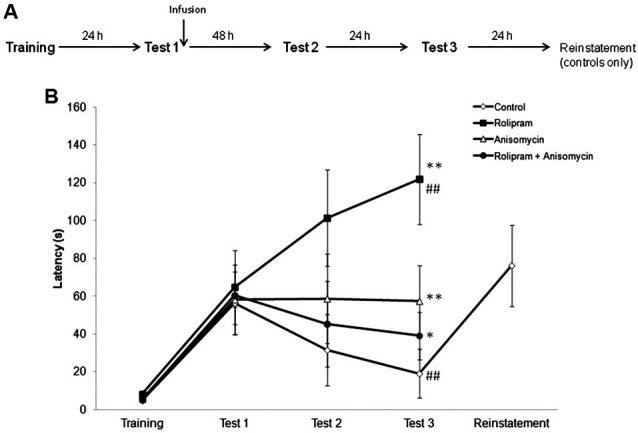
**The PDE4 inhibitor rolipram switches memory for IA from extinction to strengthening when given into the hippocampus immediately after retrieval**. Rats were given an IA training trial followed 24 h later by a retrieval session (Test 1), which also served as an extinction training trial. Immediately after Test 1, animals were infused into the dorsal hippocampus with vehicle (*N* = 9), rolipram (7.5 μg/side, *N* = 10), anisomycin (80.0 μg/side, *N* = 9), or rolipram combined with anisomycin (*N* = 10). Retention test trials were carried out 48 (Test 2) and 72 h (Test 3) after Test 1. Vehicle-treated rats received a mild reminder footshock (0.2 mA) immediately upon stepping down on Test 3, and were given an additional test trial 24 h later (“Reinstatement”). **(A)** Schematic diagram showing the experimental design. **(B)** Rats given vehicle showed extinction of IA memory, whereas rolipram-treated rats showed memory strengthening, across test trials. The protein synthesis inhibitor anisomycin prevented both extinction and rolipram-induced strengthening; * *P* < 0.05, ** *P* < 0.01 compared to the vehicle group within the same test trial; ^##^ *P* < 0.01 compared to Test 1 within the same group.

In the first experiment, we examined the effect of an intrahippocampal administration of rolipram immediately after IA memory retrieval, using a protocol that induces extinction in control rats (Vianna et al., 2001). The experimental design is shown in Figure 1A. Rats were trained in IA and underwent a retrieval session (Test 1, which also acted as extinction training) 24 h later. Immediately after retrieval, animals were infused with vehicle (*N* = 9), rolipram (*N* = 10), anisomycin (*N* = 9), or rolipram combined with anisomycin (*N* = 10). Animals were tested again 48 h (Test 2) and 72 h (Test 3) after Test 1. Rats infused with vehicle also received a mild 2.0-s reminder footshock (0.2 mA) immediately upon stepping down on Test 3, and were given an additional test trial 24 h after Test 3 (“Reinstatement”), as a procedure used to confirm that the decrease in latencies across trials was due to extinction (Tronel and Alberini, 2007).

Results are shown in Figure 1B. A Kruskal-Wallis analysis of variance showed significant differences among groups in Test 3 (*H* = 17.3, df = 3, *P* < 0.01), but not in any other behavioral session (Training, *H* = 2.3, df = 3, *P* = 0.52; Test 1, *H* = 2.60, df = 3, *P* = 0.46; Test 2, *H* = 4.3, df = 3, *P* = 0.23). Further analysis with Mann-Whitney tests showed that rats given rolipram or anisomycin (*P*s < 0.01), or rolipram combined with anisomycin (*P* < 0.05) had latencies in Test 3 that were significantly higher than those in control rats given vehicle. Rats infused with rolipram alone had higher Test 3 latencies compared to rats given anisomycin or rolipram combined with anisomycin (*P*s < 0.05). In control rats given vehicle, retention test latencies progressively declined across test trials, indicating memory extinction. A Friedman test showed a significant decrease in latencies across test trials (*H* = 13.8, df = 2, *P* < 0.01). Step-down latencies in this group went back to the levels observed in Test 1 in the “Reinstatement” test trial following a reminder shock, consistent with what would be expected for memory extinction. In contrast, rats infused with rolipram showed a progressive *enhancement* of IA retention across test trials (comparison among all three test trials using a Friedman test, *H* = 9.5, df = 2, *P* < 0.01). There were no differences between test trials within the groups treated with either anisomycin or rolipram combined with anisomycin (comparison among all three test trials using a Friedman test, anisomycin, *H* = 1.4, df = 2, *P* = 0.49; rolipram plus anisomycin, *H* = 4.2, df = 2, *P* = 0.12).

These results indicate that (1) in rats trained and tested in a protocol that induces extinction, intrahippocampal rolipram caused memory strengthening rather than extinction to occur after retrieval, and (2) blocking protein synthesis in the dorsal hippocampus prevented both extinction in vehicle-treated rats and the rolipram-induced retention strengthening in animals receiving the drug.

### Memory enhancement by post-retrieval administration of rolipram requires recall

**Figure 2:**
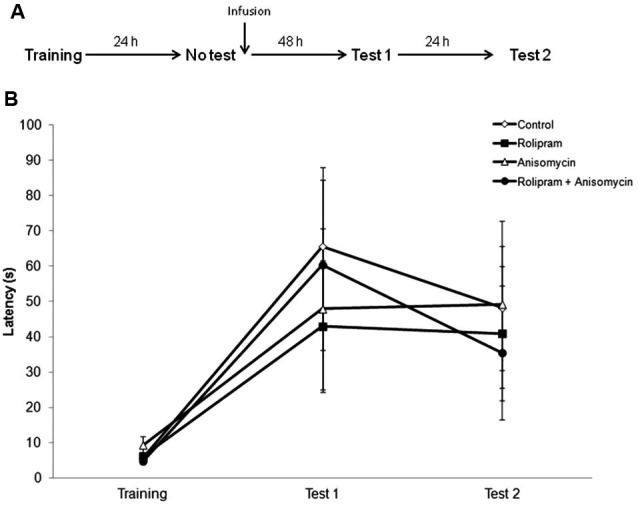
**No retrieval control**. Rats were trained in IA and 24 h later received an intrahippocampal infusion of vehicle (*N* = 13), rolipram (7.5 μg/side,* N* = 12), anisomycin (80.0 μg/side* N* = 7), or anisomycin plus rolipram (*N* = 9), in the absence of a retrieval session. Retention test trials were carried out at both 48 (Test 1) and 72 h (Test 2) after infusion. **(A)** Schematic diagram showing the experimental design. **(B)** Latencies to step-down during IA behavioral trials. There were no significant differences among groups.

The second experiment was a “no retrieval control” in which we verified whether retrieval was necessary for the memory facilitation induced by rolipram. Rats were infused with vehicle (*N* = 13), rolipram (*N* = 12), anisomycin (*N* = 7), or anisomycin plus rolipram (*N* = 9), 24 h after training in the absence of a retrieval trial (Figure 2A). Rats were tested for retention at both 48 h (Test 1) and 72 h (Test 2) after infusion. There were no significant differences among groups (Kruskal-Wallis test, Training, *H* = 2.9, df = 3, *P* = 0.41; Test 1, *H* = 1.0, df = 3, *P* = 0.80; Test 2, *H* = 2.0, df = 3, *P* = 0.56; Figure 2B). These results confirm that the drug infusion needs to be paired with retrieval in order for rolipram to enhance memory.

### Delayed post-retrieval administration of rolipram into the hippocampus does not affect memory

**Figure 3:**
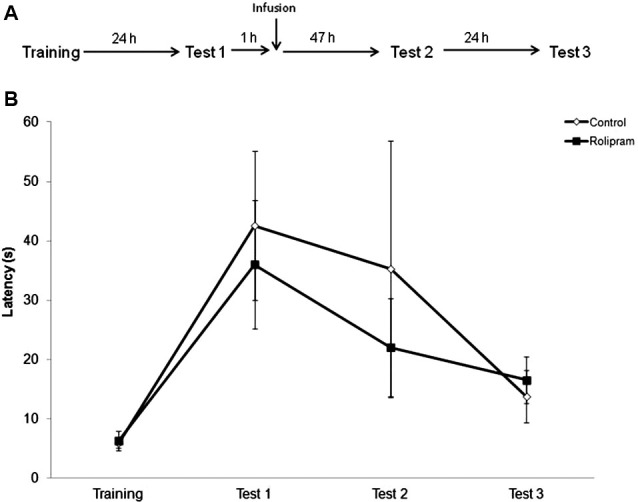
**Delayed infusion control**. Rats were given an IA training trial followed 24 h later by a retrieval/extinction session (Test 1). 1 h after Test 1, animals were infused into the dorsal hippocampus with vehicle or rolipram (7.5 μg/side,* N* = 8 rats per group). Retention test trials were carried out 48 (Test 2) and 72 h (Test 3) later. **(A)** Schematic diagram showing the experimental design. **(B)** Latencies to step-down during IA behavioral trials. There were no significant differences among groups.

Rolipram had no effect when the intrahippocampal infusion was given 1 h after retrieval measured at Test 1 (“delayed infusion control”), indicating that PDE4 inhibition can modulate memory strengthening specifically at an early time period after retrieval (Figure 3). Rats were trained and tested as in the first experiment. Mann-Whitney tests showed no significant differences between groups (Training, *P* = 0.88; Test 1, *P* = 0.72; Test 2, *P* = 0.72; Test 3, *P* = 0.42; *N* = 8 rats per group).

### The memory-enhancing effect of intrahippocampal rolipram given after retrieval lasts for at least 1 week

**Figure 4:**
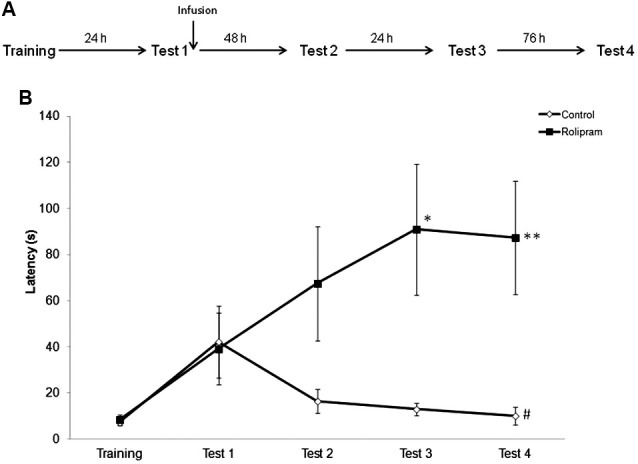
**Persistence of the memory enhancement induced by rolipram administration after retrieval**. Rats were given an IA training trial followed by retention test trials at 24 h (Test 1), 72 h (Test 2), 96 h (Test 3), and 7 days (Test 4) later. Vehicle (*N* = 10) or rolipram (7.5 μg/side,* N* = 9) was infused immediately after Test 1. **(A)** Schematic diagram showing the experimental design. **(B)** Vehicle-treated rats showed extinction of IA memory, whereas rats infused with rolipram showed memory strengthening, across test trials; * *P* < 0.05, ** *P* < 0.01 compared to the vehicle group within the same behavioral trial; ^#^ *P* < 0.05 compared to Test 1 within the same group.

In order to examine the persistence of the memory-enhancing effect of post-retrieval rolipram, rats were trained as before and tested at 24 h (Test 1), 72 h (Test 2), 96 h (Test 3), and 7 days (Test 4) later. Vehicle (*N* = 10) or rolipram (*N* = 9) was infused immediately after Test 1 (Figure 4A). Mann-Whitney tests showed significant differences between groups in Test 3 (*P* < 0.05) and Test 4 (*P* < 0.01), but not in Training (*P* = 0.97), Test 1 (*P* = 0.55), or Test 2 (*P* = 0.13). Control rats, but not rats given rolipram, showed a significant decrease in latencies across test trials (Friedman test, comparison across tests trials, *H* = 8.4, df = 3, *P* < 0.05) (Figure 4B). The results indicate that the enhancing effect of intrahippocampal rolipram given immediately after retrieval can last for at least 1 week.

### Administration of rolipram into the hippocampus immediately after training does not affect memory consolidation

**Figure 5:**
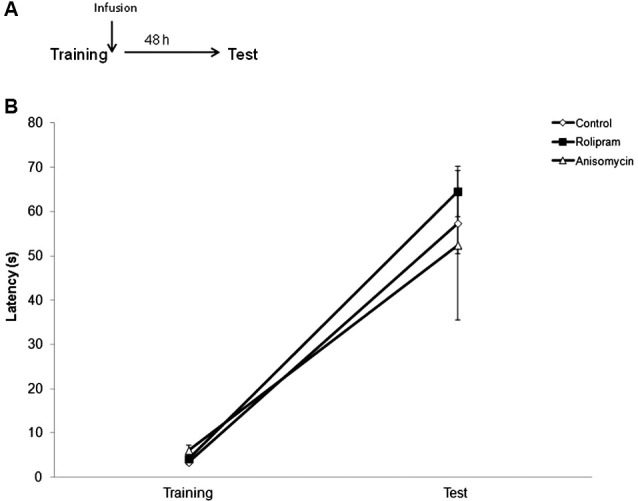
**Neither rolipram nor anisomycin affect IA memory formation when infused into the hippocampus immediately after training**. Rats were given an IA training trial followed 48 h later by a test trial. An intrahippocampal infusion of vehicle (*N* = 11), rolipram (7.5 μg/side,* N* = 12) or anisomycin (80.0 μg/side, *N* = 9) was given immediately after training. **(A)** Schematic diagram showing the experimental design. **(B)** Latencies to step-down during IA behavioral trials. There were no significant differences among groups.

In the last experiment, we verified whether rolipram and anisomycin could also affect IA memory consolidation. Vehicle (*N* = 11), rolipram (*N* = 12) or anisomycin (*N* = 9) was infused into the hippocampus immediately after training, and retention was tested 48 h later. Results are shown in Figure 5. There were no differences between groups in Training (Kruskal Wallis test, Training, *H* = 3.8, df = 2, *P* = 0.15; Test, *H* = 4.2, df = 2, *P* = 0.12). The result suggests that neither rolipram nor anisomycin affected the consolidation phase of memory when given early after training.

## Discussion

Recall of a fear-motivated memory can lead to extinction, which likely involves the creation of a second memory trace that decreases fear expression (Quirk and Mueller, 2008). Alternatively, retrieval can induce the labilization of the original memory, which again becomes sensitive to interference, a process usually referred to as reconsolidation. It has been proposed that reconsolidation can serve to maintain, update, or alter the strength of memories (Sara, 2000a,b; Amaral et al., 2008; Lee, 2008, 2010; Alberini, 2011; Alberini and Ledoux, 2013; de Oliveira Alvares et al., 2013; Reichelt and Lee, 2013).

Several studies have shown that whether a retrieved memory will undergo extinction or reconsolidation depends on the conditions under which the memory is learned and reactivated, factors that are generally manipulated experimentally by altering the training intensity, retrieval session duration, or intervals between behavioral trials (Eisenberg et al., 2003; Pedreira and Maldonado, 2003; Suzuki et al., 2004; Lee et al., 2006; Inda et al., 2011; Flavell and Lee, 2013). Here, we show that, within an experimental condition that promotes extinction in control rats, inhibiting PDE4 in the dorsal hippocampus can alter the fate of the memory towards strengthening. Both extinction and rolipram-induced strengthening depend on protein synthesis, since infusion with anisomycin blocked both processes. Control experiments omitting the first retrieval trial or using delayed and posttraining infusions indicate that the effects were not due to long-lasting drug-induced alterations in locomotion, motivation, anxiety, sensory function, or other nonspecific factors. To our knowledge, this is the first direct demonstration of a pharmacologically-inducible “switch” between memory extinction and reconsolidation.

Although most studies on reconsolidation have focused on the *disruption* of recalled memories by administration of amnestic agents, there is previous evidence that some drug treatments can *enhance* retention when paired with retrieval. Early studies showed that systemic injections of strychnine after retrieval could enhance IA memory in rats (Gordon, 1977). More recently, memories for fear conditioning in rats have been shown to be enhanced by post-retrieval administration of drugs including the protein kinase A (PKA) activator 6-BNZ-cAMP infused into the basolateral amygdala (BLA; Tronson et al., 2006), the partial *N*-methyl-D-aspartate (NMDA) receptor agonist D-cycloserine injected systemically (Lee et al., 2006), or the CB1 cannabinoid receptor antagonist AM251 infused into the dorsal hippocampus (de Oliveira Alvares et al., 2008). However, in all previous studies, the experimental conditions used were such that control rats did not show significant extinction across test trials.

Memory strengthening has been observed after either reinforced (i.e., with additional training) or non-reinforced (retrieval alone in the absence of a reinforcing stimulus) re-exposure to the learning context (Roesler et al., 1998, 2000; Quevedo et al., 1999; Lee, 2008; Roesler and Quevedo, 2009; Inda et al., 2011; Pedroso et al., 2013; Reichelt and Lee, 2013). Since strengthening depends critically on retrieval of the original memory (Roesler and Quevedo, 2009), and requires molecular mechanisms in the hippocampus that specifically underlie reconsolidation (Lee, 2008), is has been proposed that reconsolidation is the mechanism mediating strengthening (Lee, 2008; Alberini and Ledoux, 2013). Memory enhancement by repeated retrieval has been seen as a possible adaptive function of reconsolidation, since it allows relevant fear memories to be strengthened without requiring re-exposure to the original aversive learning experience (Alberini and Ledoux, 2013). It should be noted, however, that our findings do not clearly allow us to exclude the possibility that mechanisms other than reconsolidation mediate memory strengthening. One argument against the possibility of reconsolidation in this case is the fact that the latencies of animals treated with rolipram combined with anisomycin were similar between Test 2 and Test 1, suggesting that anisomycin selectively blocked the rolipram-induced enhancement without affecting the original memory. Thus, rolipram could be inducing a condition in which memory reinforcement occurs without labilization of the original memory (Osan et al., 2011; Pedroso et al., 2013). However, it should be noted that reconsolidation blockade with post-retrieval intrahippocampal anisomycin has not been consistently demonstrated in the step-down IA task (Vianna et al., 2001). Moreover, a slight trend for decreased latencies across the 3 test trials was observed in rats receiving rolipram and anisomycin, although this did not reach statistical significance. Thus, our data do not exclude the possibility that reconsolidation-like mechanisms are involved in the memory strengthening effect observed.

It has been hypothesized that high levels of attention or arousal during retrieval could reinforce the memory trace through endogenous mechanisms that might involve increased release of modulators such as catecholamines (Sara, 2000b). This possibility is consistent with the studies mentioned above showing that drugs that stimulate modulatory pathways can enhance memory when given shortly after retrieval. The findings reported by Tronson et al. (2006) showing strengthening of fear conditioning memory by a PKA activator after retrieval are particularly relevant for comparison with our present results, since PDE4 inhibitors such as rolipram enhance memory by increasing neuronal levels of cAMP, thus ultimately activating the cAMP/PKA/cAMP response-element binding protein (CREB) pathway (Barad et al., 1998; Bach et al., 1999; Bourtchouladze et al., 2003; Tully et al., 2003; Gong et al., 2004; de Lima et al., 2008). Further support for a crucial role of cAMP/PKA/CREB signaling in promoting memory strengthening upon retrieval has been provided by recent evidence that the experimentally-induced activation of amygdalar neurons expressing elevated CREB was sufficient to induce the recall of an established fear memory and promote a reconsolidation-like reorganization process leading to memory strengthening (Kim et al., 2014). The cAMP/PKA/CREB pathway is a particularly promising candidate mechanism regulating the fate of memories during retrieval, since it is crucially involved in memory formation and mediates the actions of many endogenous modulators of emotional memory, including dopamine and norepinephrine (Abel et al., 1997; Bevilaqua et al., 1997; Bach et al., 1999; Tully et al., 2003; Quevedo et al., 2004; Roesler and Schröder, 2011).

In previous studies using IA, we found that similar retrieval conditions could result in memory extinction (Luft et al., 2006), reconsolidation sensitive to impairment by mTOR inhibition (Jobim et al., 2012), or protein-synthesis dependent, retrieval-induced, memory strentgthening (Pedroso et al., 2013). However, the behavioral and neurochemical factors determining these different outcomes of retrieval remain elusive. According to the “trace dominance” model, the result of a retrieval session/extinction trial involves the sum of multiple and conflicting processes, including a competition between the original excitatory memory trace and a new inhibitory extinction trace, for the control of behavior (Eisenberg et al., 2003). More recent computational work has proposed that network dynamics can lead to strengthening without labilization, reconsolidation or extinction depending on the degree of mismatch between the original memory and the retrieval session (Osan et al., 2011). Nevertheless, the current results indicate that the definition of the dominant process during retrieval can be altered by pharmacological manipulation of the hippocampus.

The present findings indicate that PDE4 inhibition, presumably by enhancing cAMP signaling, can shift the balance between the processes occurring during retrieval, directing a recalled memory in a way that favors strengthening rather than extinction. In this sense, it is interesting to note that, in fear conditioning, some data suggest that hippocampal and prefrontal inputs converge on the amygdala, with the former driving fear expression and reconsolidation and the latter favoring extinction (Herry et al., 2008; Mamiya et al., 2009). It is possible that stimulating neuronal populations responsible for the representation of the fear memory in the hippocampus through manipulation of the AMPc/PKA/CREB cascade could shift this balance in favor of hippocampal inputs driving maintenance and strengthening of the original memory. This hypothesis should be further examined by future experiments.

Although both protein synthesis and PKA activity in the dorsal hippocampus are required for memory formation, we did not find effects of rolipram or anisomycin when infused after learning. However, previous reports have indicated that intra-hippocampal anisomycin can impair IA memory consolidation when given before or 3 h after, but not immediately after training (Quevedo et al., 1999). Also, drugs acting on the PKA pathway have been shown to influence IA memory consolidation only when infused into the hippocampus 3 h posttraining (Bevilaqua et al., 1997). Thus, the reason for the lack of effect of rolipram and anisomycin in this case is likely to be related to temporal factors, and does not imply that IA consolidation is independent from protein synthesis.

In conclusion, we provide evidence suggesting that the behavioral outcome of the recall of an established memory can be pharmacologically switched from extinction towards strengthening through a purely pharmacological intervention, by pairing retrieval with PDE4 inhibition in the dorsal hippocampus. These findings may contribute to our understanding of the factors governing memory modifications induced by recall.

## Conflict of interest statement

The authors declare that the research was conducted in the absence of any commercial or financial relationships that could be construed as a potential conflict of interest.
